# Prognostic and Immunological Role of Key Genes of Ferroptosis in Pan-Cancer

**DOI:** 10.3389/fcell.2021.748925

**Published:** 2021-10-13

**Authors:** Zhi-Zhou Shi, Hao Tao, Ze-Wen Fan, Sheng-Jie Song, Jie Bai

**Affiliations:** Medical School, Kunming University of Science and Technology, Kunming, China

**Keywords:** ferroptosis, SLC7A11, GPX4, AIFM2, pan-cancer

## Abstract

Solute carrier family 7 member 11 (SLC7A11), glutathione peroxidase 4 (GPX4), and apoptosis inducing factor mitochondria associated 2 (AIFM2) are the key regulators in ferroptosis. However, the expression patterns and prognostic roles of these genes in pan-cancer are still largely unclear. The expression patterns and prognostic roles of SLC7A11, GPX4, and AIFM2 and the relationships between the expression levels of these genes and immune infiltration levels in pan-cancer were analyzed by using TIMER, gene expression profiling interactive analysis (GEPIA), Oncomine, and Kaplan–Meier databases. Our results showed that both SLC7A11 and GPX4 were overexpressed in colorectal cancer, and SLC7A11 was overexpressed in lung cancer. High levels of SLC7A11 and AIFM2 were significantly linked with the shortened disease-free survival and overall survival (OS) in adrenocortical carcinoma (ACC), respectively. And high expression of SLC7A11, GPX4, and AIFM2 were significantly correlated with the shortened OS of acute myeloid leukemia patients. In esophageal carcinoma (ESCA), GPX4 expression was significantly associated with the infiltration of macrophage and myeloid dendritic cell, and AIFM2 expression was significantly associated with the infiltration of CD4^+^ T cell. Importantly, GPX4 expression was positively correlated with the expression levels of monocyte markers (CD14 and CD115) and M2 macrophage markers (VSIG4 and MS4A4A) both in ESCA and in head and neck squamous cell carcinoma (HNSC). In summary, SLC7A11, GPX4, and AIFM2 are dysregulated in many types of cancers, and are candidate prognostic biomarkers for many types of cancers, and can be used to evaluate the infiltration of immune cells in tumor tissues.

## Introduction

Ferroptosis is a new form of regulated cell death induced by iron-dependent lipid peroxidation and with the characteristic of oxidative perturbations of the intracellular microenvironment (Chen et al., 2021). A recent study suggested that ferroptosis might be developed as a natural and promising therapy against cancer (Wang et al., 2021a). However, up to now, the expression pattern and prognostic value of key regulators of ferroptosis especially the correlation between the expression levels of these genes and immune infiltration in pan-cancer are largely unclear.

Solute carrier family 7 member 11 (SLC7A11), the catalytic subunit of a heterodimeric cystine/glutamate antiporter (System Xc^–^), transports extracellular cystine into cells for glutathione biosynthesis and is identified as a key regulator of ferroptosis. Knockout or knockdown of SLC7A11 markedly suppressed the growth, survival, and tumor formation of cancer cells by inducing ferroptosis (Daher et al., 2019; Lim et al., 2019; Wang et al., 2020). Overexpression of SLC7A11 was detected in KRAS-mutant lung adenocarcinoma (LUAD) and positively correlated with cancer progression. Genetic depletion or pharmacological inhibition with sulfasalazine or HG106 markedly induced the cell death of KRAS-mutant cancer cells *in vitro* and tumor growth suppression *in vivo* (Hu et al., 2020). Immunotherapy-activated CD8^+^ T cells secreted interferon gamma (IFNγ), and then downregulated SLC7A11 in tumor cells and finally promoted cancer cell lipid peroxidation and ferroptosis (Wang et al., 2019). Combination of radiotherapy and immunotherapy could enhance the lipid oxidation and ferroptosis of cancer cells by synergistically targeting and suppressing SLC7A11 (Lang et al., 2019).

Glutathione peroxidase 4 (GPX4), an antioxidant enzyme and a key regulator of ferroptosis, could catalyze and reduce lipid peroxides (Yang et al., 2014). Knockdown of GPX4 could induce the ferroptosis of oral cancer cells (Fukuda et al., 2021). Inhibiting GPX4 by resibufogenin (RB) induced ferroptotic cancer cell death and suppressed tumor growth *in vivo* (Shen et al., 2021). GPX4 was reported to be involved in the metformin, miR-324-3p, and loss of PTPN18 or SRSF9 induced ferroptosis (Deng et al., 2021; Hou et al., 2021; Wang et al., 2021b,c). Apoptosis inducing factor mitochondria associated 2 (AIFM2, also known as PRG3 or FSP1), a traditional apoptotic regulator, was identified as a new ferroptosis regulator and found to block the RSL3-, sorafenib-, and erastin-induced ferroptosis of cancer cells, and inhibition of AIFM2-dependent pathway enhanced the antitumor activity of sorafenib in mouse model (Bersuker et al., 2019; Doll et al., 2019; Dai et al., 2020).

In the present study, we comprehensively analyzed the mRNA levels of SLC7A11, GPX4, and AIFM2, and the correlations between the mRNA levels of these three genes and prognosis in pan-cancer using databases including Oncomine, gene expression profiling interactive analysis (GEPIA), TIMER, and Kaplan–Meier Plotter. We further investigated the relationships between the expression levels of SLC7A11, GPX4, and AIFM2 and immune infiltration levels in pan-cancer by using the TIMER database.

## Materials and Methods

### Kaplan–Meier Plotter Database Analysis

Kaplan--Meier Plotter^1^ was used to evaluate the prognostic roles of SLC7A11, GPX4, and AIFM2 in 21 different cancers using 7,462 cancer samples in overall survival (OS) analysis and 4,420 cancer samples in relapse-free survival (RFS) analysis (Nagy et al., 2021). The best cutoff was automatically selected by the database program.

### TIMER Database Analysis

TIMER database^2^ was used to analyze the expression patterns of SLC7A11, GPX4, and AIFM2, and the correlation between the expression levels of SLC7A11, GPX4, or AIFM2 and immune infiltration, and the correlation between the expression levels of SLC7A11, GPX4, or AIFM2 and markers of different subsets of immune cells [including CD8^+^ T cells, CD4^+^ T cells, B cells, tumor-associated macrophages (TAMs), monocytes, M1 macrophages, M2 macrophages, neutrophils, dendritic cells (DCs), natural killer (NK) cells, and several types of T cells including T helper 1 (Th1), Th2, follicular helper T (Tfh), Th17, regulatory T (Tregs), and exhausted T cells] in pan-cancer including more than 10,000 samples from The Cancer Genome Atlas (TCGA) (Li et al., 2016, 2017; Li T. et al., 2020).

### Gene Expression Profiling Interactive Analysis Database Analysis

The GEPIA database^3^ was used to evaluate the prognostic roles of SLC7A11, GPX4, and AIFM2 in pan-cancer including 9,736 tumors and 8,587 normal samples from the TCGA and the GTEx projects (Tang et al., 2017).

### Oncomine Database Analysis

The mRNA expression levels of SLC7A11, GPX4, and AIFM2 in several cancers such as breast cancer, colorectal cancer (CRC), and so on were analyzed by using Oncomine database^4^ (Rhodes et al., 2007).

### Statistical Analysis

The expression patterns and prognostic roles of SLC7A11, GPX4, and AIFM2 were analyzed by using TIMER, GEPIA, Oncomine, and Kaplan–Meier databases. The correlation between the expression levels of SLC7A11, GPX4, or AIFM2 and immune infiltration, and the correlation between the expression levels of SLC7A11, GPX4, or AIFM2 and markers of different subsets of immune cells were analyzed by using TIMER database. *p*-Value < 0.05 was regarded as statistically significant.

## Results

### The mRNA Expression Levels of Solute Carrier Family 7 Member 11, Glutathione Peroxidase 4, and Apoptosis Inducing Factor Mitochondria Associated 2 in Pan-Cancer

**FIGURE 1:**
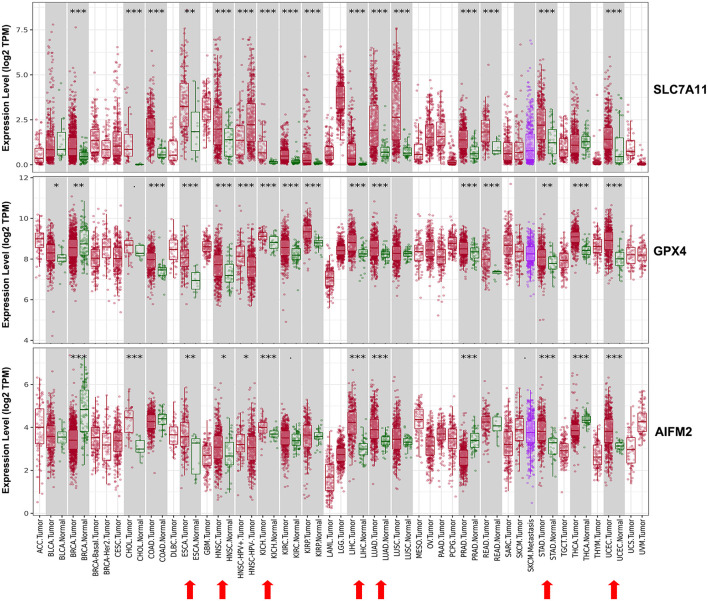
Expression patterns of SLC7A11, GPX4, and AIFM2 in pan-cancer. The expression patterns of SLC7A11, GPX4, and AIFM2 were analyzed by using TIMER database. ^∗^*p* < 0.05; ^∗∗^*p* < 0.01; ^∗∗∗^*p* < 0.001.

We evaluated the mRNA expression levels of SLC7A11, GPX4, and AIFM2 in TCGA RNA-seq data by analyzing TIMER database. The results in Figure 1 showed that all of the three genes were significantly higher in esophageal carcinoma (ESCA, head and neck squamous cell carcinoma (HNSC), kidney chromophobe (KICH), liver hepatocellular carcinoma (LIHC), LUAD, stomach adenocarcinoma (STAD), and uterine corpus endometrial carcinoma (UCEC). Very importantly, both SLC7A11 and GPX4 were overexpressed in CRC including colon adenocarcinoma (COAD) and rectum adenocarcinoma (READ) and upregulated in pan-kidney cohort including kidney renal clear cell carcinoma (KIRC), KICH, and kidney renal papillary cell carcinoma (KIRP) comparing with adjacent normal tissues. We also found that SLC7A11 was overexpressed both in LUAD and lung squamous cell carcinoma (LUSC).

**FIGURE 2:**
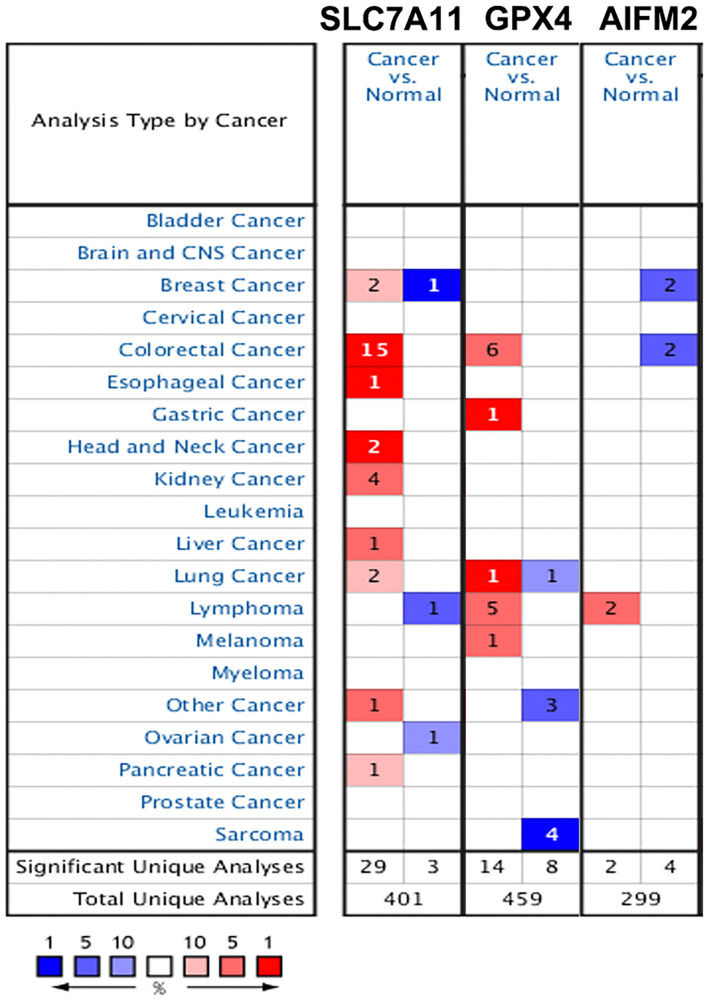
Expression patterns of SLC7A11, GPX4, and AIFM2 in pan-cancer. The expression patterns of SLC7A11, GPX4, and AIFM2 were analyzed by using Oncomine database. %, gene rank percentile (%).

We also analyzed the mRNA levels of SLC7A11, GPX4, and AIFM2 in pan-cancer using Oncomime database. The results showed that SLC7A11 was higher in CRC, esophageal cancer, head and neck cancer, kidney cancer, liver cancer, lung cancer, pancreatic cancer, and uterus cancer, and lower in lymphoma and ovarian cancer (Figure 2 and Supplementary Table 1). The expression of GPX4 was higher in CRC, gastric cancer, lymphoma and melanoma, and lower in sarcoma. AIFM2 was overexpressed in lymphoma and under-expressed in breast cancer and CRC. Interestingly, both SLC7A11 and GPX4 were overexpressed in CRC (in 15 and 6 datasets, respectively, Figure 2 and Supplementary Table 1), and these findings suggested that anti-ferroptosis might be the characteristic of CRC cells.

### Prognostic Values of Solute Carrier Family 7 Member 11, Glutathione Peroxidase 4, and Apoptosis Inducing Factor Mitochondria Associated 2 in Pan-Cancer

**FIGURE 3:**
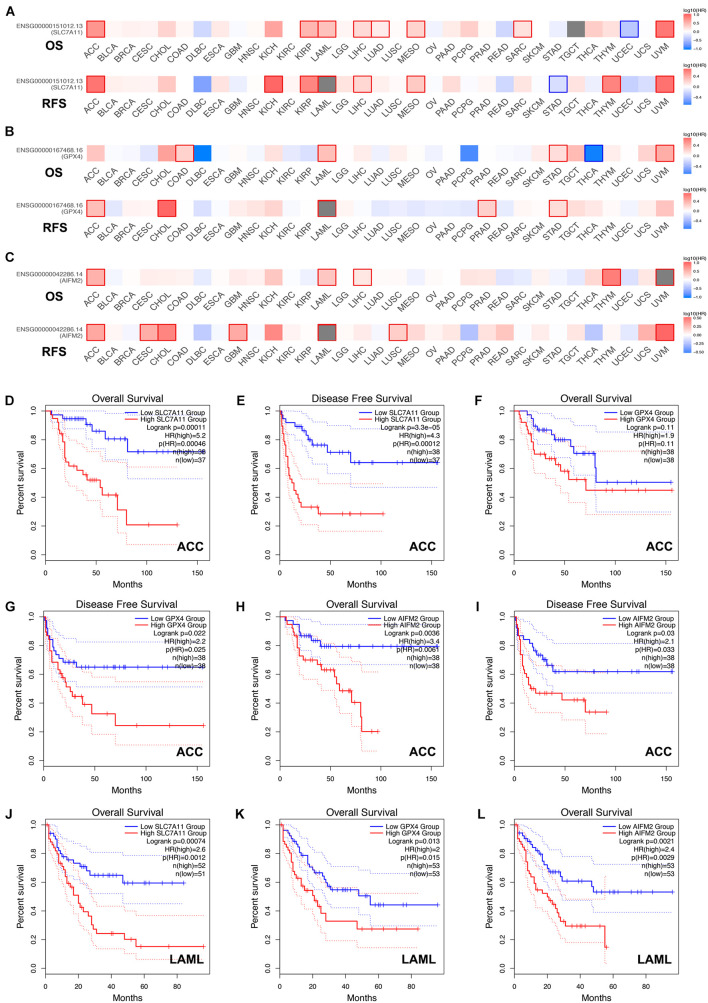
Prognostic roles of SLC7A11, GPX4, and AIFM2 in pan-cancer. **(A–C)** Prognostic pattern of SLC7A11, GPX4, and AIFM2 in pan-cancer were analyzed by using GEPIA database. **(D–I)** The correlations between SLC7A11, GPX4, or AIFM2 and OS or DFS of ACC patients were evaluated by using GEPIA database. **(J–L)** The correlations between SLC7A11, GPX4, or AIFM2 and OS of LAML patients were evaluated by using GEPIA database.

**FIGURE 4:**
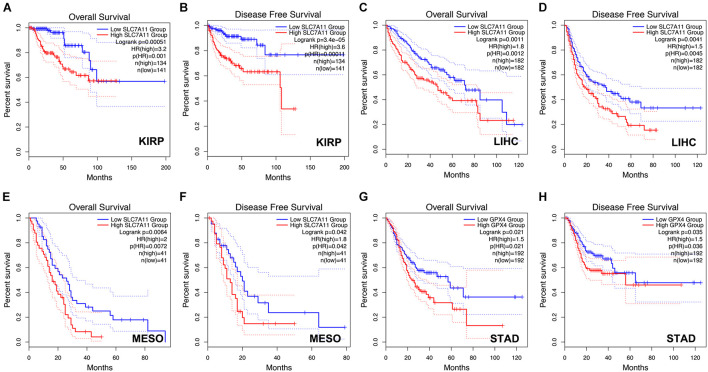
Prognostic role of SLC7A11 in KIRP, LIHC, and MESO and prognostic role of GPX4 in STAD. **(A–F)** The correlations between SLC7A11 and OS or DFS of KIRP, LIHC, and MESO patients were evaluated by using GEPIA database. **(G,H)** The correlations between GPX4 and OS or DFS of STAD patients were evaluated by using GEPIA database.

Next, we analyzed the prognostic values of SLC7A11, GPX4, and AIFM2 in pan-cancer using GEPIA database. We found that high expression of SLC7A11, GPX4, and AIFM2 were significantly linked with the shortened disease-free survival in adrenocortical carcinoma (ACC), respectively, and high expression of SLC7A11 and AIFM2 were significantly associated with shortened OS in ACC respectively (Figures 3A–I). And high expression of SLC7A11, GPX4, and AIFM2 were significantly correlated with the shortened OS of acute myeloid leukemia (LAML) patients (Figures 3A–C,J–L). Interestingly, we further found that high expression of SLC7A11 were significantly associated with shortened OS and disease-free survival of KIRP, LIHC, and MESO (mesothelioma), and high expression of GPX4 was significantly correlated with shortened OS and disease-free survival of STAD (Figures 4A–H).

We also analyzed the prognostic values of SLC7A11, GPX4, and AIFM2 in pan-cancer using Kaplan–Meier database. High expression of SLC7A11 was significantly correlated with the poor OS and RFS in bladder carcinoma, breast cancer, kidney renal papillary cell carcinoma, liver hepatocellular carcinoma, and pancreatic ductal adenocarcinoma (Table 1). Importantly, the higher mRNA levels of SLC7A11, GPX4, and AIFM2 were all significantly linked with the good OS in UCEC, and both higher expression of SLC7A11 and AIFM2 were significantly correlated with the poor OS and RFS in liver hepatocellular carcinoma (Table 1). Different from SLC7A11 in bladder carcinoma, the high expression of AIFM2 was significantly associated with the good OS and RFS (Table 1).

**TABLE 1:** Prognostic values of SLC7A11, GPX4, and AIFM2 in pan-cancer in Kaplan–Meier database.

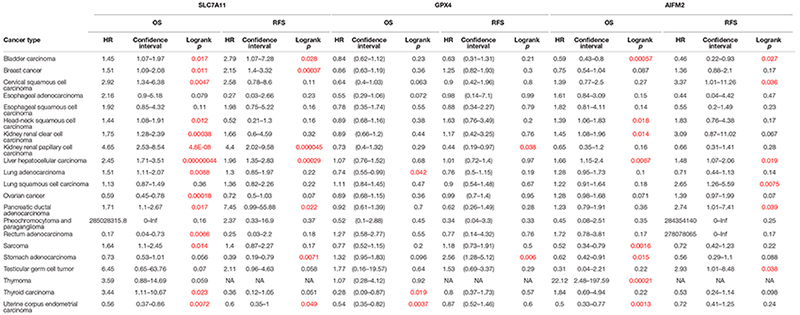

*Red: significant (*p* < 0.05).*

### Correlation Between the Expression Levels of Solute Carrier Family 7 Member 11, Glutathione Peroxidase 4, or Apoptosis Inducing Factor Mitochondria Associated 2 and Immune Infiltration in Pan-Cancer

**FIGURE 5:**
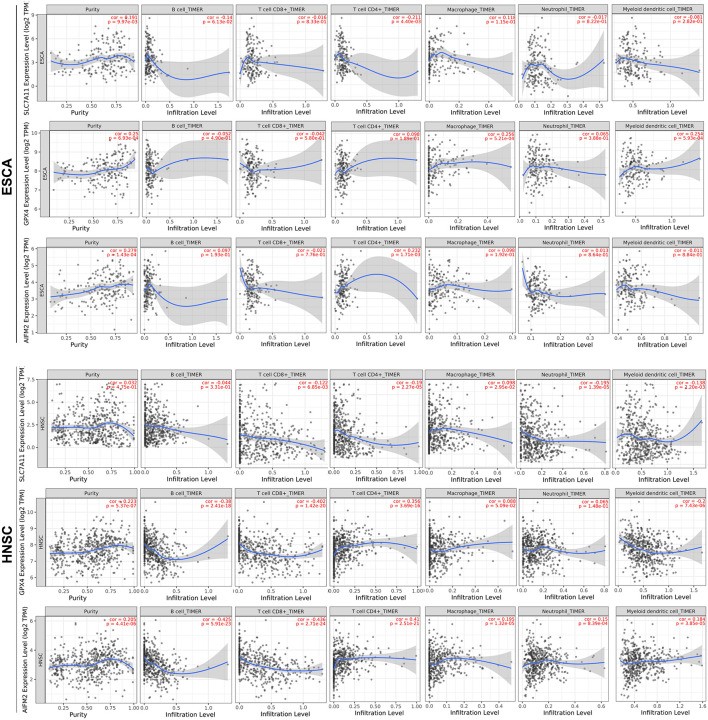
Correlation analysis of SLC7A11, GPX4, or AIFM2 and infiltration levels of immune cells in ESCA and HNSC. The correlation analysis of SLC7A11, GPX4, or AIFM2 and infiltration levels of immune cells in ESCA and HNSC was performed by using TIMER database.

We further analyzed the correlation between the expression levels of SLC7A11, GPX4, or AIFM2 and immune infiltration in ESCA, HNSC, COAD, READ, STAD, and LUAD in which at least two of the three ferroptosis-associated genes were overexpressed. We observed that high expression levels of GPX4 and AIFM2 were significantly associated with tumor purity in ESCA (*R* = 0.25, *p* = 6.93e-04; *R* = 0.279, *p* = 1.43e-04) and in HNSC, respectively (*R* = 0.223, *p* = 5.37e-07; *R* = 0.205, *p* = 4.41e-06; Figure 5). In ESCA, the level of SLC7A11 was significantly associated with the infiltration level of CD4^+^ T cell (*R* = −0.211, *p* = 4.40e-03), and the level of GPX4 was significantly correlated with the infiltration level of macrophage (*R* = 0.256, *p* = 5.21e-04) and DC (*R* = 0.254, *p* = 5.93e-04), and the level of AIFM2 was markedly linked with the infiltration level of CD4^+^ T cell (*R* = 0.232, *p* = 1.71e-03). In HNSC, the level of GPX4 was significantly correlated with the infiltration level of B cell (*R* = −0.38, *p* = 2.41e-18), CD8^+^ T cell (*R* = −0.402, *p* = 1.42e-20), CD4^+^ T cell (*R* = 0.356, *p* = 3.69e-16), and DC (*R* = 0.2, *p* = 7.43e-06), and the level of AIFM2 was significantly associated with the infiltration level of B cell (*R* = −0.425, *p* = 5.91e-23), CD8^+^ T cell (*R* = −0.436, *p* = 2.71e-24), and CD4^+^ T cell (*R* = 0.41, *p* = 2.51e-21; Figure 5).

In COAD, the expression of SLC7A11 was significantly correlated with the infiltration level of CD8^+^ T cell (*R* = 0.229, *p* = 1.32e-04). In READ, the expression of SLC7A11 were significantly associated with the infiltration level of neutrophil (*R* = 0.295, *p* = 4.73e-03). In STAD, the expression of SLC7A11 was significantly associated with the infiltration level of CD4^+^ T cell and macrophage, respectively (*R* = −0.259, *p* = 3.13e-07; *R* = −0.212, *p* = 3.09e-05). In LUAD, the expression of SLC7A11 was significantly associated with the infiltration level of DC (*R* = −0.206, *p* = 4.01e-06), and the expression of AIFM2 was significantly associated with the infiltration level of macrophage, neutrophil, and DC, respectively (*R* = −0.248, *p* = 2.34e-08; *R* = −0.201, *p* = 6.89e-06; *R* = −0.218, *p* = 1.01e-06; Supplementary Figures 1, 2).

### Correlation Between the Expression Levels of Solute Carrier Family 7 Member 11, Glutathione Peroxidase 4, or Apoptosis Inducing Factor Mitochondria Associated 2 and Markers of Different Subsets of Immune Cells in Pan-Cancer

By analyzing the gene expression data of immune markers in the TIMER database, we investigated the association between the levels of SLC7A11, GPX4, and AIFM2 and the status of tumor-infiltrating immune cells in ESCA, HNSC, COAD, READ, STAD, and LUAD. The CD8^+^ T cells, CD4^+^ T cells, B cells, TAMs, monocytes, M1 macrophages, M2 macrophages, neutrophils, DCs, NK cells, and several types of T cells including Th1, Th2, Tfh, Th17, Tregs, and exhausted T cells were analyzed in our study. The correlation analysis in the present study was adjusted for purity because of the influence of tumor purity on the immune infiltration.

In ESCA, GPX4 expression was significantly correlated with the expression of markers of immune cells (at least two significant correlated markers in one cell type) such as monocyte markers, CD14 (*R* = 0.299, *p* = 4.59e-05) and CD115 (*R* = 0.252, *p* = 6.57e-04); TAM markers, CCL2 (*R* = 0.341, *p* = 2.84e-06) and IL10 (*R* = 0.288, *p* = 8.65e-05); M1 macrophage markers, iNOS (*R* = −0.29, *p* = 7.63e-05), IL6 (*R* = 0.293, *p* = 6.57e-05), and CD64 (*R* = 0.29, *p* = 7.74e-05); M2 macrophage markers, VSIG4 (*R* = 0.307, *p* = 2.73e-05) and MS4A4A (*R* = 0.307, *p* = 2.73e-05); and DC markers, BDCA-1 (*R* = 0.244, *p* = 9.68e-04) and BDCA-4 (*R* = 0.26, *p* = 4.14e-04; Table 2). In HNSC, GPX4 expression was significantly correlated with the expression of markers of immune cells (at least two significant correlated markers in one cell type) such as B cell markers, CD19 (*R* = 0.207, *p* = 3.51e-06) and CD27 (*R* = 0.248, *p* = 2.52e-08); monocyte markers, CD14 (*R* = 0.205, *p* = 4.74e-06) and CD115 (*R* = 0.236, *p* = 1.14e-07); M1 macrophage markers, iNOS (*R* = 0.293, *p* = 3.65e-11) and CD64 (*R* = 0.282, *p* = 1.79e-10); M2 macrophage markers, VSIG4 (*R* = 0.217, *p* = 1.12e-06) and MS4A4A (*R* = 0.248, *p* = 2.44e-08); and T cell exhaustion markers, CTLA4 (*R* = 0.223, *p* = 5.83e-07) and TIM-3 (*R* = 0.259, *p* = 5.35e-09; Table 2). Interestingly, both in ESCA and HNSC, the expression of GPX4 was significantly positively associated with the monocyte markers, CD14 and CD115, and the M2 macrophage markers, VSIG4 and MS4A4A.

**TABLE 2:** Correlation between the expression levels of SLC7A11, GPX4, or AIFM2 and markers of different subsets of immune cells in ESCA and HNSC.

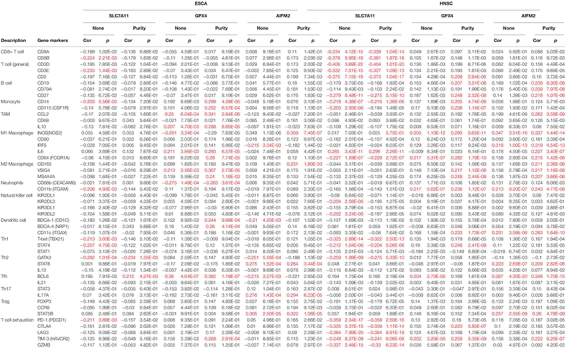

*Red: | R| ≥ 0. 2 and *p* < 0.05.*

In HNSC, the expression of SLC7A11 was negatively correlated with CD8^+^ T cell markers, CD8A (*R* = −0.339, *p* = 1.04e-14) and CD8B (*R* = −0.381, *p* = 1.87e-18); T cell (general) markers, CD3D (*R* = −0.404, *p* = 1.01e-20), CD3E (*R* = −0.327, *p* = 1.09e-13), and CD2 (*R* = −0.373, *p* = 1.04e-17); B cell marker, CD27 (*R* = −0.275, *p* = 5.15e-10); monocyte marker, CD14 (*R* = −0.216, *p* = 1.36e-06); M1 macrophage marker, CD64 (*R* = −0.229, *p* = 2.72e-07); Th1 markers, T-bet (*R* = −0.322, *p* = 2.50e-13) and STAT4 (*R* = −0.204, *p* = 5.06e-06); Th2 marker, GATA3 (*R* = −0.275, *p* = 5.33e-10); and T cell exhaustion markers, PD-1 (*R* = −0.358, *p* = 2.50e-16), CTLA4 (*R* = −0.339, *p* = 1.11e-14), LAG3 (*R* = −0.384, *p* = 8.91e-19), TIM-3 (*R* = −0.241, *p* = 6.06e-08), and GZMB (*R* = −0.33, *p* = 6.23e-14; Table 2). In HNSC, the expression of AIFM2 was significantly correlated with the expression of markers of immune cells (at least two significant correlated markers in one cell type) such as B cell, CD19 (*R* = 0.239, *p* = 8.30e-08), CD79A (*R* = 0.239, *p* = 7.97e-08), and CD27 (*R* = 0.218, *p* = 1.07e-06); M1 macrophage markers, iNOS (*R* = 0.337, *p* = 1.44e-14), IRF5 (*R* = 0.319, *p* = 4.54e-13), IL6 (*R* = 0.227, *p* = 3.40e-07), and CD64 (*R* = 0.215, *p* = 1.42e-06); and M2 macrophage markers, CD163 (*R* = 0.211, *p* = 2.36e-06), VSIG4 (*R* = 0.217, *p* = 1.16e-06), and MS4A4A (*R* = 0.207, *p* = 3.66e-06; Table 2). Very interestingly, the expression of AIFM2 was significantly associated with the expression of STAT6 and STAT5B both in ESCA and HNSC.

In READ, SLC7A11 expression was significantly correlated with the expression of markers of immune cells (at least two significant correlated markers in one cell type) such as monocyte markers, CD14 (*R* = −0.286, *p* = 6.48e-04) and CD115 (*R* = −0.2, *p* = 1.84e-02); and DC markers, BDCA-1 (*R* = −0.232, *p* = 6.04e-03) and BDCA-4 (*R* = 0.256, *p* = 2.31e-03; Supplementary Table 2). GPX4 expression was associated with the expression of marker immune cells (at least two significant correlated markers in one cell type) such as monocyte markers, CD14 (*R* = 0.339, *p* = 4.58e-05) and CD115 (*R* = 0.269, *p* = 1.36e-03); M1 macrophage markers, iNOS (*R* = −0.242, *p* = 4.08e-03) and CD64 (*R* = 0.334, *p* = 6.00e-05); and DC markers, BDCA-1 (*R* = 0.218, *p* = 9.79e-03) and BDCA-4 (*R* = −0.205, *p* = 1.53e-02; Supplementary Table 2).

In LUAD, SLC7A11 expression was significantly correlated with the expression of markers of immune cells (at least two significant correlated markers in one cell type) such as M1 macrophage markers, CD80 (*R* = −0.217, *p* = 1.20e-06) and IRF5 (*R* = −0.246, *p* = 3.25e-08); and DC markers, BDCA-1 (*R* = −0.346, *p* = 2.62e-15) and BDCA-4 (*R* = −0.227, *p* = 3.48e-07). GPX4 expression was significantly correlated with the expression of markers of immune cells (at least two significant correlated markers in one cell type) such as M1 macrophage markers, CD80 (*R* = −0.229, *p* = 2.85e-07) and CD64 (*R* = −0.204, *p* = 5.23e-06); and M2 macrophage markers, VSIG4 (*R* = −0.216, *p* = 1.36e-06) and MS4A4A (*R* = −0.234, *p* = 1.44e-07; Supplementary Table 2).

In COAD and STAD, there were no correlations between SLC7A11/GPX4/AIFM2 with the expression of markers of immune cells (at least two significant correlated markers in one cell type).

Very interestingly, the expression of SLC7A11 was significantly positively associated with the level of STAT1 in COAD, READ, and STAD (*R* = 0.256, *p* = 1.77e-07; *R* = 0.337, *p* = 5.07e-05; *R* = 0.21, *p* = 3.78e-05). The expression of GPX4 was significantly negatively associated with the level of STAT3 in COAD, READ, and LUAD (*R* = −0.217, *p* = 1.02e-05; *R* = −0.288, *p* = 5.98e-04; *R* = −0.229, *p* = 2.77e-07).

## Discussion

Recent studies reported that therapy-resistant tumor cells were vulnerable to ferroptosis; therefore, ferroptosis induction will be a potential therapeutic method for tumors especially drug-resistant tumors (Li B. et al., 2020; Jiang et al., 2021). Our study found that both SLC7A11 and GPX4 were overexpressed in CRC, and SLC7A11 was highly expressed in lung cancer by analyzing TIMER and Oncomine databases. These findings were consistent with the published literatures. SLC7A11 and GPX4 were highly expressed in CRC cell lines and CRC tissues compared with paired normal tissues, and downregulation of SLC7A11 by talaroconvolutin or downregulation of GPX4 by apatinib or inactivation of GPX4 by resibufogenin could induce ferroptosis of CRC cells (Yagublu et al., 2011; Ma et al., 2015; Xia et al., 2020; Shen et al., 2021; Tian et al., 2021). SLC7A11 was overexpressed in non-small cell lung cancer (NSCLC) tissues and its overexpression was significantly correlated with poor prognosis of NSCLC patients (Ji et al., 2018). Inhibition of SLC7A11 by sulforaphane or YTHDC2 (a m6A reader) induced the ferroptosis of lung cancer cells and suppressed the progression of lung cancer (Hu et al., 2020; Iida et al., 2021; Ma et al., 2021). Interestingly, our results also showed that AIFM2 was overexpressed in ESCA and HNSC; however, up to now, the roles and mechanisms of AIFM2 in the progression of ESCA and HNSC were still unknown.

By analyzing the GEPIA database, we found that high expression of SLC7A11, GPX4, and AIFM2 were correlated with the shortened disease-free survival in ACC respectively, and high expression of SLC7A11 and AIFM2 were significantly associated with shortened overall survival in ACC respectively. Previous studies have reported that human ACC cells NCI-H295R cell line was sensitive to ferroptosis induction, and the mRNA level of SLC7A11 was higher in ACCs than in normal adrenal glands (nAGs) and its low expression was significantly associated with good OS of ACC patients (Belavgeni et al., 2019; Weigand et al., 2020). However, the functional roles and regulatory mechanisms of SLC7A11, GPX4, and AIFM2 in the ferroptosis of ACC cells were still unclear. Our study also revealed that high expression of SLC7A11, GPX4, and AIFM2 were significantly correlated with the lower overall survival of LAML. A previous study reported that combination of APR-246 and inactivation of SLC7A11/GPX4 could synergistically promote the ferroptosis of AML cells both in *in vivo* and *ex vivo* experiments (Birsen et al., 2021). However, how SLC7A11 and GPX4 were regulated in LAML and whether AIFM2 was involved in the regulation of ferroptosis of AML cells were still largely unknown. Therefore, future studies should focus on the roles of ferroptosis in the progression of ACC and LAML, especially the regulatory mechanisms of SLC7A11 or GPX4 or AIFM2 in ferroptosis of ACC and LAML cells.

Although a previous study reported that GPX4 could prevent Treg cells from lipid peroxidation and ferroptosis (Xu et al., 2021), whether SLC7A11, GPX4, and AIFM2 participated in the regulation of ferroptosis of other immune cells and tumor progression is still largely unknown. Our study demonstrated that in ESCA, GPX4 expression was significantly associated with the infiltration of macrophage and myeloid DC, and AIFM2 expression was significantly associated with the infiltration of CD4^+^ T cell. These findings suggested that GPX4 and AIFM2 might be involved in the progression of esophageal cancer probably by regulating the ferroptosis of different immune cells. Our results also showed that the expression levels of GPX4 and AIFM2 were correlated with the infiltration of B cell, CD8^+^ T cell, and CD4^+^ T cell in HNSC, respectively. This suggested that GPX4 and AIFM2 played important roles in regulating tumor immunity by affecting the ferroptosis of B cell, CD8^+^ T cell, and CD4^+^ T cell in HNSC. Our study also reported that the expression of AIFM2 was significantly correlated with the expression of markers of B cell including CD19, CD79A, and CD27.

Interestingly, our study also indicated that GPX4 expression was positively correlated with the expression levels of monocyte markers including CD14 and CD115 and M2 macrophage markers including VSIG4 and MS4A4A both in ESCA and in HNSC. Hsieh et al. reported that zero-valent-iron nanoparticle (ZVI-NP) could promote the shift from pro-tumor M2 macrophages to anti-tumor M1 macrophages and finally inhibit the tumor progression (Hsieh et al., 2021). Our results suggested that activation of M2 macrophages in ESCA and HNSC might be correlated with high expression of GPX4.

## Conclusion

Our results suggest that SLC7A11, GPX4, and AIFM2 are dysregulated in many types of cancers, and are candidate prognostic biomarkers for many types of cancers, and can be used to evaluate the infiltration of immune cells in tumor tissues. Future studies should focus on the regulation of ferroptosis of tumor cells and immune cells in different types of cancers.

## Data Availability Statement

The datasets presented in this study can be found in online repositories. The names of the repository/repositories and accession number(s) can be found in the article/Supplementary Material.

## Author Contributions

Z-ZS and HT analyzed the data. Z-ZS, HT, Z-WF, S-JS, and JB wrote the manuscript. All authors contributed to the article and approved the submitted version.

## Conflict of Interest

The authors declare that the research was conducted in the absence of any commercial or financial relationships that could be construed as a potential conflict of interest.

## Publisher’s Note

All claims expressed in this article are solely those of the authors and do not necessarily represent those of their affiliated organizations, or those of the publisher, the editors and the reviewers. Any product that may be evaluated in this article, or claim that may be made by its manufacturer, is not guaranteed or endorsed by the publisher.

## Footnotes

1
http://kmplot.com/analysis/

2
http://timer.cistrome.org

3
http://gepia2.cancer-pku.cn/#index

4
www.oncomine.org/resource/main.html
