# Supplementary figures and images for: Prognostic and Immunological Role of Key Genes of Ferroptosis in Pan-Cancer
Source: Front Cell Dev Biol. 2021 Oct 13;9:748925. doi: 10.3389/fcell.2021.748925 (PMC8548644; doi:10.3389/fcell.2021.748925)

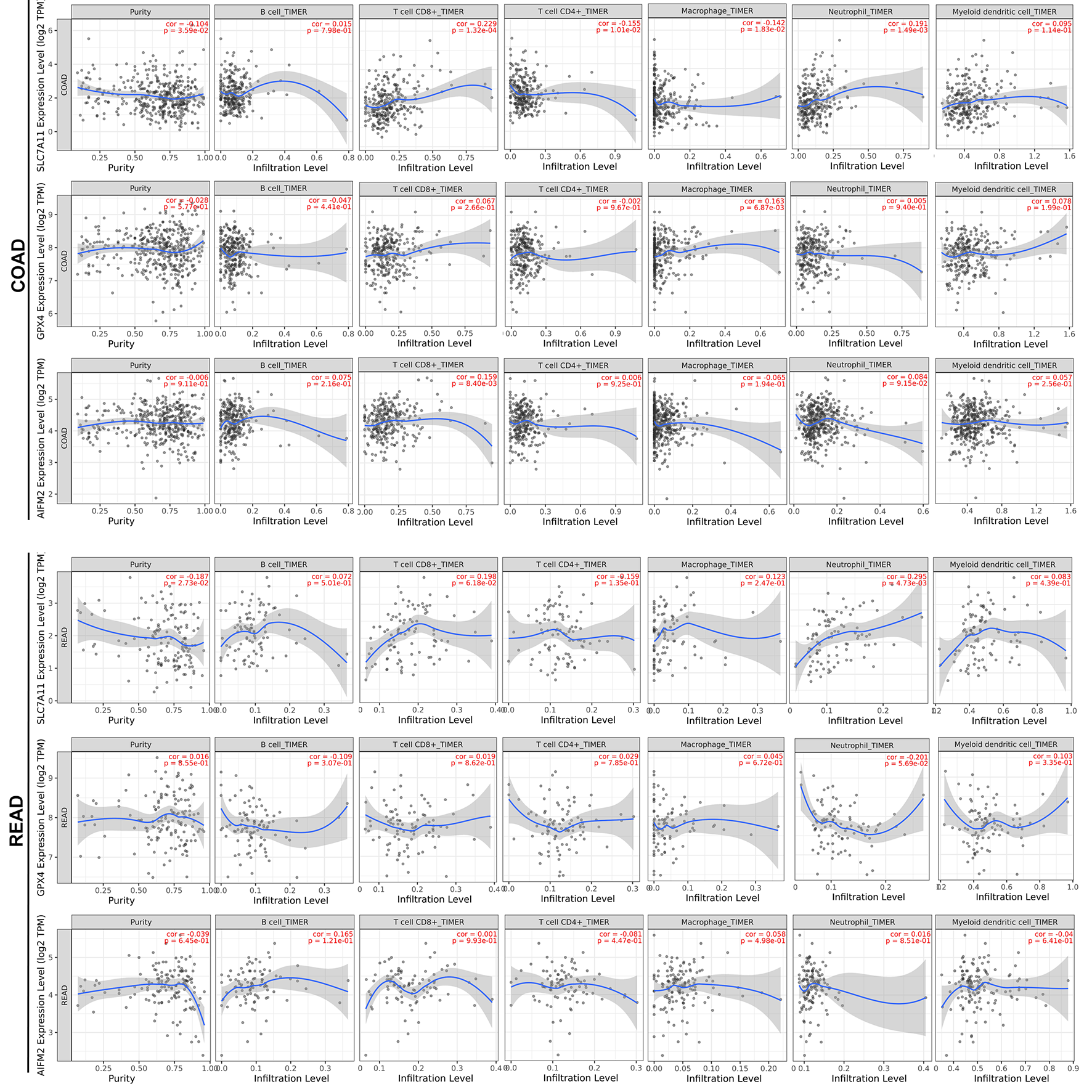

Supplement: Supplementary file 1 [file Image_1.TIF]

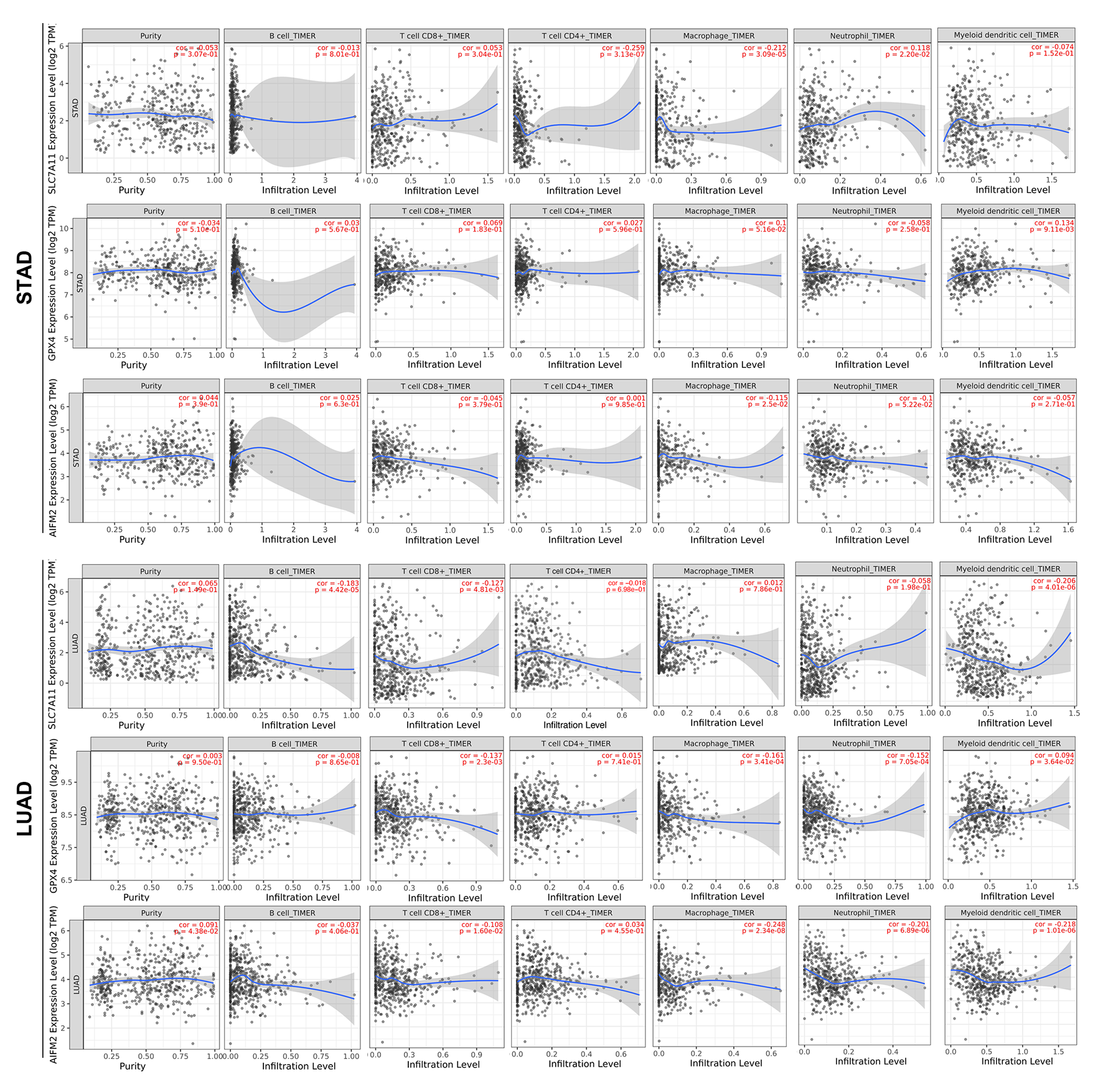

Supplement: Supplementary file 2 [file Image_2.TIF]
